# Comprehensive untargeted metabolomics of Lychnnophorinae subtribe (Asteraceae: Vernonieae) in a phylogenetic context

**DOI:** 10.1371/journal.pone.0190104

**Published:** 2018-01-11

**Authors:** Maria Elvira Poleti Martucci, Benoit Loeuille, José Rubens Pirani, Leonardo Gobbo-Neto

**Affiliations:** 1 University of São Paulo, School of Pharmaceutical Sciences of Ribeirão Preto, Ribeirão Preto, Sao Paulo, Brazil; 2 University of São Paulo, Institute of Biosciences, Department of Botany, São Paulo, Sao Paulo, Brazil; University of San Agustin, PHILIPPINES

## Abstract

Members of the subtribe Lychnophorinae occur mostly within the *Cerrado* domain of the Brazilian Central Plateau. The relationships between its 11 genera, as well as between Lychnophorinae and other subtribes belonging to the tribe Vernonieae, have recently been investigated upon a phylogeny based on molecular and morphological data. We report the use of a comprehensive untargeted metabolomics approach, combining HPLC-MS and GC-MS data, followed by multivariate analyses aiming to assess the congruence between metabolomics data and the phylogenetic hypothesis, as well as its potential as a chemotaxonomic tool. We analyzed 78 species by UHPLC-MS and GC-MS in both positive and negative ionization modes. The metabolic profiles obtained for these species were treated in *MetAlign* and in *MSClust* and the matrices generated were used in SIMCA for hierarchical cluster analyses, principal component analyses and orthogonal partial least square discriminant analysis. The results showed that metabolomic analyses are mostly congruent with the phylogenetic hypothesis especially at lower taxonomic levels (*Lychnophora* or *Eremanthus*). Our results confirm that data generated using metabolomics provide evidence for chemotaxonomical studies, especially for phylogenetic inference of the Lychnophorinae subtribe and insight into the evolution of the secondary metabolites of this group.

## Introduction

Vernonieae contains 21 currently recognized subtribes [1–4]. Among these, the subtribe Lychnophorinae is nearly endemic to Brazil [1,2,5,6]. Additionally, it contains 18 genera and ca. 120 species [1,2,5,6]. Most species are restricted to *campo rupestre* (literally rocky fields) in the highlands of southeastern and northeastern Brazil and to the *Cerrado* domain (Brazilian savanna). From a phytochemical point of view, these species exhibit high diversity of compounds. Flavonoids and terpenoids have been extensively identified; reviewing the absence of diterpenes and the extensive reports of sesquiterpene lactones (SLs) from the germacranolides (specifically germacrolides and heliangolides sub-types) and guaianolides types [7–10]. In a review of American Vernonieae [11, 12], was proposed a morphological definition of Lychnophorinae that is rather consistent with the recent phylogeny of the American Vernonieae [3]. This latter work also indicates the inclusion in Lychnophorinae of two small subtribes (Centratherinae, Sipolisiinae) and three small genera (*Albertinia*, *Blanchetia* and *Gorceixia*) previously placed in subtribes Vernoniinae and Piptocarphinae.

However, the subtribe holds a high proportion of monotypic genera (42%), reflecting how poorly understood relationships between the genera of the group are. The generic limits of the two diversified genera (ca. 50%), *Eremanthus* and *Lychnophora*, are controversial [3,5]. *Haplostephium* [10,13,14,15], *Lychnophoriopsis* [5] and *Paralychnophora* [6,16] have been variously recognized at a generic level. Also, several species of Lychnophorinae have an uncertain generic position (in *Eremanthus*, *Lychnophora* or *Piptolepis*) [17,18]. In a phylogeny of Lychnophorinae based on DNA sequences and morphological data [5], the richest genus of the subtribe was *Lychnophora*, with 32 described species, but it emerged as polyphyletic, involving three main lineages not closely related. Most of the obtained clades were associated with some existing generic concepts and they can be defined by a simple combination of morphological characteristics *Albertinia*, *Blanchetia* and *Gorceixia* are the earliest diverging lineages, whereas *Eremanthus* and *Lychnophora strict sensu* emerged as the most derived clade, which contains half of the species of the subtribe. However, relationships between the remaining clades persist partially unresolved, and some generic concepts require further refinement.

Metabolomics involves the study of metabolites in biological systems and aims to establish chemical or metabolite differences among samples, relating them to genotypes of each species [19,20]. The untargeted metabolomics analysis aims to establish global metabolite signals measured by one or more analytical platforms [21]. During the last decades, these approaches have benefited by improvements in hyphenated mass spectrometry (UHPLC-MS and GC-MS), which has become highly sensitive and allows a more complete chemical profile of the analyzed samples [20].

Metabolomics approaches may be a valuable tool for chemotaxonomic characterization and providing primary data for phylogenetic inference. In a recent study, we used metabolomics aimed at higher plant chemotaxonomical purposes, showing the applicability of untargeted analysis to help with taxonomical classifications [22].

In this study, we report the use of a comprehensive untargeted metabolomics approach, employing UHPLC(DAD)-MS(ESI-ORBITRAP) and GC-MS(EI) data combined, followed by multivariate analyses. Thereafter, we compared our results to the phylogenetic hypothesis proposed by Loeuille et al. (2015b). Finally, we evaluated the possibility of employment of untargeted metabolomics as a chemotaxonomic tool for Lychnophorinae subtribe classification.

## Materials and methods

### Plant material

Seventy-eight species (Supporting Material) belonging to the Lychnophorinae subtribe were analyzed in this study. Collection and access of samples from the Brazilian Genetic Heritage was authorized by IEF and CNPq/CGen (010091/2011-4) respectively. The samples used for the metabolomics approach were the same used in phylogenetic analyses by Loeuille et al. (2015b). In this study, similar with that of Loeuille et al. (2015b), only one specimen of each species was analyzed for the purpose of mutual comparison. Furthermore, there are substantial evidences that there is no significant chemical qualitative variation between individuals from the same population of plants from this subtribe [5,7,8,23]. In addition, most of the species studied are micro-endemics with very few populations, such a scenario offers a low probability for infraspecific chemical qualitative variation. One replicate of each species was sampled for both UHPLC-MS and GC-MS analyses. To avoid analytical variations, the analyses were performed injecting 10 samples/batch and the injection of all samples was performed within a four days interval. To detect eventual variations, the same sample was analyzed before and after each batch. Eventual deviations in the internal standard retention times and areas in all the chromatograms were also checked.

### Instrumentation

The UHPLC-MS and UHPLC-HCD MS/MS experiments were performed using an *Accela* UHPLC apparatus with a diode array detector (*Accela*) coupled to an ESI-Orbitrap mass spectrometer *Exactive Plus* (Thermo Scientific). The GC-MS experiments were performed using GC coupled to an EI mass spectrometer QP2010 Shimadzu.

#### UHPLC-MS and UHPLC-HCD MS/MS analyses

UHPLC-MS and UHPLC-HCD MS/MS analyses were performed using a core shell column (Kinetex 1.7 μm XB-C18, 150 X 2.1 mm, Phenomenex) connected to a guard cartridge of the same material. Separation was performed at a flow rate of 400 μL.min^-1^ and a gradient of H_2_O-HCO_2_H (0.1%) (v/v) (A) and CH_3_CN (B) as mobile phases; the elution profile was: 0–2 min, 5% B; 2–30 min, 5–100% B; 30–34 min (column washing), 100% B; 34–37 min, 100–5% B; 37–40 min (column equilibration), 5% B. The oven temperature was set at 45°C. The DAD detector was set to record between 200–600 nm and chromatograms were registered at 254 nm, 270 nm and 330 nm. The column effluent was analyzed by ESI-MS (resolution of 70,000) and ESI-HCD MS/MS (resolution of 35,000) in both positive and negative ionization modes, all simultaneously. The mass spectra were acquired and processed using the software provided by the manufacturer. UHPLC-MS total ion current (TIC) chromatograms were recorded between *m/z* 150 and 1,200 and the following mass spectrometer parameters were maintained as the same in all analyses: 1.0 microscans per second; an automatic gain control (AGC) target, 1.0e6; maximum inject time, 100 ms; sheath gas flow rate, 30; auxiliary gas flow rate, 10; sweep gas flow rate, 11; capillar temperature, 320°C; spray voltage in positive ionization mode, 3.6 kV; spray voltage in negative ionization mode, 3.2 kV; S-lens RF level, 50; and HCD, normalized collision energy (NCE) 35.0 eV. N_2_ was used as the drying, nebulizer and fragmentation gas.

#### GC-MS analyses

Analyses by GC-MS were performed in split injection mode at 260°C, using a DB-5MS capillary column (J&W Agilent) of 30 m X 0.25 mm, and film thickness 0.25 μm, with He (79.7 kPa) as a carrier gas at a flow rate of 1.3 mL.min^-1^. An electron ionization mass spectrometer (EI-MS) detector was operated under an ion source temperature of 250°C, a trap emission current of 60 μA and a 70 eV ionization energy. The global run time was recorded in full scan mode between *m/z* 50–500 and a scanning ratio of 0.30 scan.s^-1^. The GC oven temperature was initially 100°C, then linearly rose by 3°C.min^-1^ to 300°C during 90 min.

#### Sample preparation for UHPLC-MS and UHPLC-HCDMS/MS analyses

The leaves of each single plant were dried under air circulation (35°C, 24 h) and powdered using liquid N_2_. The samples were prepared using 10.0 mg of dried powder in a glass vial and extracted with 1.0 mL of a solution of MeOH-H_2_O (7:3, v/v) in an ultrasonic bath for 10 min. Each extract was subjected to a clean-up with 500 μL of hexane. An internal standard of hydrocortisone (10.0 μg.mL^-1^) was added to the extract. Finally, each extract was filtered in a 0.20 μm PTFE membrane and 5.0 μL were injected.

#### Sample preparation for GC-MS analyses

The leaves were dried and powdered as described above. The samples were prepared using 30.0 mg of dried powder in a glass vial and extracted with 2.0 mL of CH_2_Cl_2_ in an ultrasonic bath for 30 min. Each extract was transferred to a glass vial and the solvent was evaporated. Prior to analyses, the dried extract was suspended with CH_2_Cl_2_ to a concentration of 10.0 mg.mL^-1^.

#### Untargeted data processing and multivariate analysis

Mass signals (*m/z*) from the raw data files were automatically extracted and aligned by *MetAlign* (Rikilt, Institute of Food Safety) [24], resulting in 1,061 mass signals (GC-MS) at ion intensity higher than 5,000; and 36,861 and 24,482 mass signals in both positive and negative electrospray ionization mode (UHPLC-MS), respectively, at ion intensity higher than 10^5^. Mass signals were subsequently re-grouped using *MSClust* (Netherlands Genomics Initiative/Netherlands Organization for Scientific Research) [25], resulting in 73 (GC-MS), 2,972 (UHPLC-MS negative ionization mode) and 2,974 (UHPLC-MS positive ionization mode) reconstructed spectra, in a total of 6,019. Multivariate analyses (PCA and HCA) were performed using SIMCA P 13.0.3.0 (Umetrics AB Malmö, Sweden), after submitting data to both Pareto scaling and log transformation of metabolite signal intensities. HCA was performed using Euclidean distances. Subsequently, orthogonal partial least square discriminant analysis (OPLS-DA) was performed using SIMCA P 13.0.3.0 (Umetrics AB Malmö, Sweden) in accordance with the groups obtained from the PCA and HCA. The parameters used for OPLS-DA were the same as for PCA and HCA.

#### Chromatographic peak identification

Discriminant variables were provided by OPLS-DA and a variable importance plot (VIP) provided variables important for discrimination of each class. Then, discriminant compounds were identified according to the UV spectrum and molecular formulae calculated from accurate mass measurements, both obtained from UHPLC-UV-MS analyses. These UV spectra were used to suggest secondary metabolite classes corresponding to each peak, followed by screening against the molecular formulae in the Scifinder and Dictionary of Natural Products databases. In addition, fragmentation data obtained by UHPLC-UV-HCD MS/MS analyses, as well as comparison with authentic standards whenever possible, were also used for structure elucidations and to confirm the peak assignments. Finally, identified discriminant compounds were compared with the phytochemistry previously reported for the Lychnophorinae subtribe.

## Results and discussion

### Multivariate analysis

Aqueous-methanol extracts were prepared from dried leaves and UHPLC-MS based metabolic fingerprinting was performed for all species, in both positive and negative electrospray ionization (ESI) modes. In a similar way, dichloromethane extracts were prepared from dried leaves and GC-MS-based metabolic fingerprinting was performed for all species, in an electron ionization (EI) mode. The data obtained in both positive and negative electrospray ionization (ESI) modes and the data obtained in an electron ionization (EI) mode were processed separately by *MetAlign* (Rikilt, Institute of Food Safety) [24] and reconstructed by *MSClust* (Netherlands Genomics Initiative/Netherlands Organization for Scientific Research) [25] followed by multivariate analysis with combined data from UHPLC-MS and GC-MS in SIMCA P 13.0.3.0 (Umetrics AB Malmö, Sweden).

**Fig 1:**
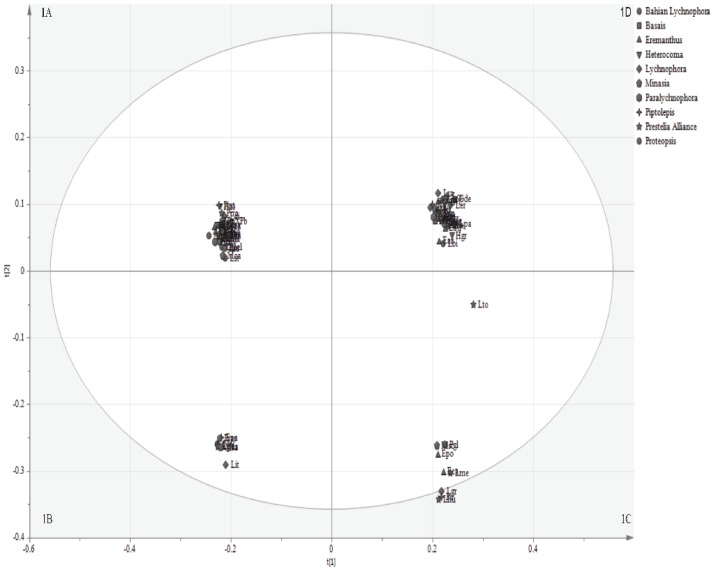
Score scatter plots of principal component analysis (t1 versus t2) of 78 analyzed species from the Lychnophorinae subtribe. Based on metabolic profiling obtained in LC-MS, in both positive and negative electrospray ionization modes, and in GC-MS. The obtained groups were respectively assigned 1A, 1B, 1C and 1D.

**Fig 2:**
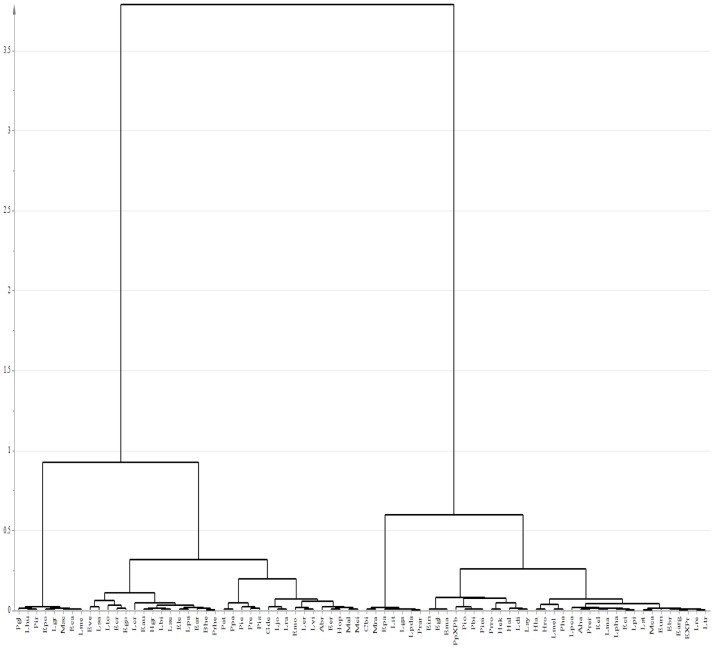
Hierarchical cluster analysis of 78 analyzed species from the Lychnophorinae subtribe. Based on metabolic profiling obtained in LC-MS, in both positive and negative electrospray ionization modes, and in GC-MS.

The principal component analysis (PCA) (Fig 1) and hierarchical cluster analysis (HCA) (Fig 2) of 78 species showed segregation into four groups and these groups were respectively assigned 1A, 1B, 1C and 1D (Table 1). None of the groups corresponded to clades of the phylogeny proposed by Loeuille et al. (2015b). However, it is possible to note that most of the species of *Eremanthus* and *Lychnophora strict sensu* were found in the groups (1A) and (1D). These groups comprised 17 among 20 analyzed species from *Eremanthus* and 16 among 23 analyzed species from *Lychnophora*.

**Table 1:** Species belonging to groups formed in PCA (Fig 1) and HCA (Fig 2).

Group	Species^a^
1A	Ein, Egl, Ema, PpXPb, Pio, Pbi, Pim, Prro, Hek, Hal, Ldi, Lsy, Hla, Hro, Lmel, Pha, Lpca, Aha, Prer, Eel, Lma, Lpha, Eci, Lpi, Lst, Mca, Eun, Ebr, Earg, EXPr, Lre, Ltr
1B	Cbi, Mra, Epa, Lit, Lga, Lpda, Prar
1C	Pgl, Lhu, Pir, Epo, Lgr, Msc, Eca, Lme, Lto
1D	Eve, Lsa, Ecr, Ego, Lcr, Eau, Hgr, Lbi, Lse, Eer, Ele, Lpa, Ear, Bhe, Prhe, Pat, Ppa, Pie, Pre, Pis, Gde, Ljo, Lra, Emo, Ler, Lvi, Abr, Hop, Mal, Mci

^a^Species (*Anteremanthus hatschbachii*—Aha), (*Albertinia brasiliensis*–Abr), (*Blanchetia heterotricha*—Bhe), (*Chronopappus bifrons*—Cbi), (*Eremanthus arboreus*—Ear), (*E*. *argenteus*—Earg), (*E*. *auriculatus*—Eau), (*E*. *brevifolius*- Ebr), (*E*. *capitatus*- Eca), (*E*. *cinctus*—Eci), (*E*. *crotonoides*–Ecr), (*E*. *elaeagnus*—Eel), (*E*. *erythropappus*—Eer), (*E*. *glomerulatus*—Egl), (*E*. *goyazensis*–Ego), (*E*. *incanus*—Ein), (*E*. *leucodendron*- Ele), (*E*. *mattogrossensis*- Ema), (*E*. *mollis*- Emo), (*E*. *pabstii*—Epa), (*E*. *polycephalus*—Epo), (*Eremanthus* sp. X *Paralychnophora reflexoauriculata* (G.M. Barroso)—EXPr), (*E*. *uniflorus*—Eun), (*E*. *veadeiroensis*—Eve), (*Gorceixia decurrens*—Gde), (*Heterocoma albida*—Hal), (*H*. *ekmanianum*—Hek), (*H*. *gracilis*- Hgr), (*H*. *lanuginosa*—Hla), (*H*. *robinsoniana*- Hro), (*Hololepis pedunculata*—Hop), (*Lychnophora bishopii*—Lbi), (*L*. *crispa*- Lcr), (*L*. *diamantinana*- Ldi), (*L*. *ericoides*—Ler), (*L*. *gardneri*- Lga), (*L*. *granmogolensis*—Lgr), (*L*. *humillima*- Lhu), (*L*. “*itacambirensis*” sp. ined.,—Lit), (*L*. “*jolyana*” sp. ined,—Ljo), (*L*. *markgravii*—Lma), (*L*. *mellobarretoi*—Lme), (*L*. *“mellosilvae”* sp. ined—Lmel), (*L*. *passerina*—Lpa), (*L*. *pinaster*—Lpi), (*L*. *ramosissima*—Lra), (*L*. *regis*—Lre), (*L*. *salicifolia*—Lsa), (*L*. *santosii* -Lst), (*L*. *sellowii*-Lse), (*L*. *syncephala*—Lsy), (*L*. *tomentosa*—Lto), (*L*. *triflora*—Ltr), (*L*. *villosissima*—Lvi), (*Lychnophoriopsis candelabrum*—Lpca), (*L*. *damazioi*—Lpda), (*L*. *hatschbachii* -Lpha), (*Minasia alpestris*—Mal), (*M*. *cabralensis*—Mca), (*M*. *“cipoensis*” sp. ined,—Mci), (*M*. *scapigera*—Msc), (*M*. *ramosa*- Mra), (*Paralychnophora atkinsiae*—Pat), (*P*. *bicolor*—Pbi), (*P*. *glaziouana*- Pgl), (*P*. *harleyi*—Pha), (*P*. *patriciana*—Ppa), (*P*. *patriciana* X *P*. *bicolor* -PpXPb), (*P*. *reflexoauriculata* Pre), (*Piptolepis ericoides*—Pie), (*P*. *oleaster*—Pio), (*P*. *monticola*- Pim), (*P*. “*riparia*” sp. ined.,—Pir), (*P*. *schultziana*- Pis), (*Prestelia eriopus*—Prer), (*Prestelia* “*robusta*” sp. ined,—Prro), (*Proteopsis argentea*—Prar), (*P*. “*hermogenesii*” sp. ined.,—Prhe).

Regarding *Heterocoma*, *H*. *albida* (Hal), *H*. *robinsoniana* (Hro) and *H*. *lanuginose* (Hla) were found in the group (1A), whereas *H*. *ekmaniana* (Hek) and *H*. *gracilis* (Hgr) belonged to the group (1D). The hierarchical cluster analysis (HCA) of 78 species (Fig 2) showed *H*. *albida*, *H*. *ekmaniana*, *H*. *robinsoniana* and *H*. *lanuginose* within the same group, and only *H*. *gracilis* belonging to another group. Regarding *Lychnophoriopsis*, *L*.*hatschbachii* (Lpha) and *L*. *candelabrum* (Lpca) belonged to group (1D), whereas *L*. *damazioi* (Lpda) belonged to group (1B); which was also observed with HCA (Fig 2). These results corroborated that *Lychnophoriopsis* should be treated as a synonym of *Lychnophora*, and that *L*. *damazioi* is not closely related to *Lychnophora*, as proposed by Loeuille et al. (2015b).

*Paralychnophora atkinsiae* (Pat), *P*. *patriciana* (Ppa) and *P*. *reflexoauriculata* (Pre) emerged within the same group (1D), and *P*. *bicolor* (Pbi) and the hybrid *P*. *patriciana* X *P*. *bicolor* (PpXPb) belonged to group (1A), which was also confirmed by HCA (Fig 2). About *Piptolepis*, *P*. *monticola* (Pmo) and *P*. *oleaster* (Pio) belonged to group (1A); while *P*. *ericoides* (Pie) and *P*. *schultziana* (Pis) belonged to group (1D), as was also noted with HCA (Fig 2).

It is noteworthy that multivariate analysis showed higher robustness (R2 cumulative = 0.444 and Q2 cumulative = 0.325) when UHPLC-MS and GC-MS data were analyzed together. Therefore, combining UHPLC-MS and GC-MS allows analysis of polar and nonpolar metabolites, respectively, providing a more widespread and robust metabolomics analysis. The resulting multivariate analysis using only the UHPLC-MS data (R2 cumulative = 0.291 and Q2 cumulative = 0.167) is shown in the Supporting Material (Figures A and B in S1 File).

**Fig 3:**
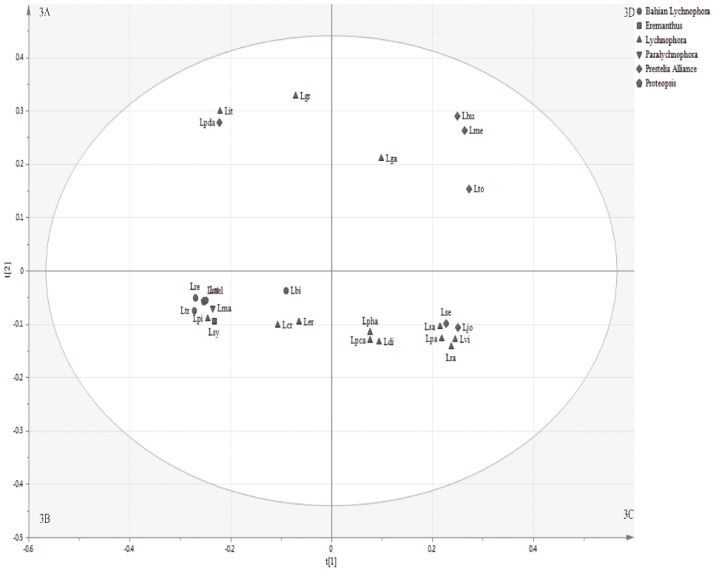
Score scatter plots of principal component analysis (t1 versus t2) of *Lychnophora* species. Based on metabolic profiling obtained in LC-MS, in both positive and negative electrospray ionization modes, and in GC-MS. The obtained groups were respectively assigned 3A, 3B, 3C and 3D.

*Lychnophora* is one of the richest genera of the subtribe; therefore, it was analyzed separately by multivariate analysis with combined data from UHPLC-MS and GC-MS in SIMCA P 13.0.3.0 (Umetrics AB Malmö, Sweden). The multivariate analysis results for the *Lychnophora* species (Fig 3) are comparable with the phylogeny proposed by Loeuille et al. (2015b). The groups formed in PCA (Fig 3) were respectively assigned 3A, 3B, 3C and 3D. Also, the hierarchical cluster analysis (HCA) of *Lychnophora* species is shown in in the Supporting Material (Figure C in S1 File).

Species classified in the *Prestelia* Alliance clade were found in the group (3D), except for *Lychnophoriopsis damazioi* (Lpda), which was included in group (3A) and *Lychnophora* “jolyana” sp. ined. (Ljo) and *L*. *sellowii* (Lse), which were in group (3C), as most of the *Lychnophora* species. Among species classified as *Lychnophora* and belonging to the group (3C), *L*. *passerina* (Lpa), *L*. *ramosissima* (Lra), *L*. *salicifolia* (Lsa) and *L*. *villosissima* (Lvi) emerged very close to each other, while a little further away, it was possible to observe *L*. *diamantinana* (Ldi), *Lychnophoriopsis candelabrum* (Lpca) and *L*. *hatschbachii* (Lpha). On the other hand, *L*. *crispa* (Lcr), *L*. *ericoides* (Ler) and *L*. *pinaster* (Lpi) were found in the group (3B), while *L*. *granmogolensis* Lgr) and *L*. *itacambirensis* (Lit) belonged to group (3A) and *L*. *gardneri* (Lga) was the only species in group (3D).

All species of Bahian *Lychnophora* belonged in the same group (3B), with *Lychnophora santosii* (Lst), *L*. *regis* (Lre) and *L*. *triflora* (Ltr) very close to each other, and *L*. *bishopii* (Lbi) a little farther away. It should be noted that Bahian *Lychnophora* comprised a number of *Lychnophora* species and *Eremathus leucodendron* (Ele), and they are restricted to the *Campos Rupestres* of the *Chapada Diamantina*, the northern sector of the *Espinhaço* range of mountains, in the State of Bahia, Eastern Brazil they are morphologically distinct from the rest of *Lychnophora* species [5].

**Fig 4:**
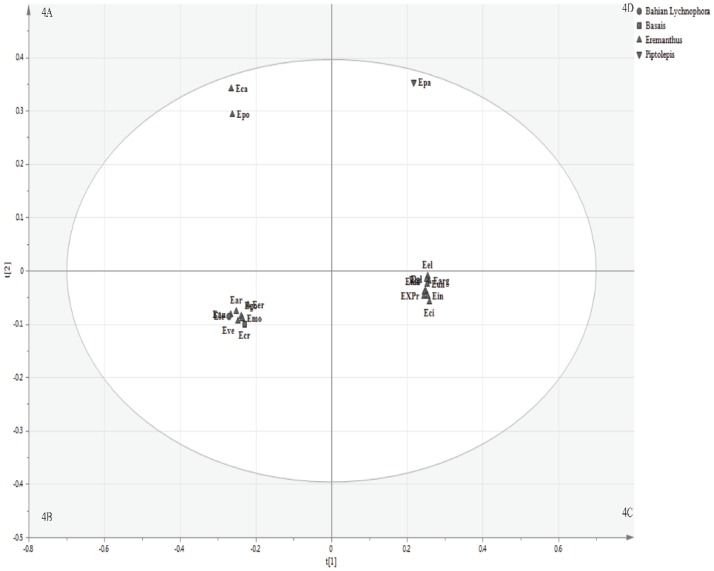
Score scatter plots of principal component analysis (t1 versus t2) of *Eremanthus* species. Based on metabolic profiling obtained in LC-MS, in both positive and negative electrospray ionization (ESI) modes, and in GC-MS. The obtained groups were respectively assigned 4A, 4B, 4C and 4D.

*Eremanthus*, the second speciose genus of the subtribe, was further analyzed separately by multivariate analysis with combined data from UHPLC-MS and GC-MS in SIMCA P 13.0.3.0 (Umetrics AB Malmö, Sweden). Multivariate analysis results for *Eremanthus* species (Fig 4) were comparable with the phylogeny proposed by Loeuille et al. (2015b). Species classified as *Eremanthus* segregated into four groups, which were respectively assigned 4A, 4B, 4C and 4D. Also, the hierarchical cluster analysis (HCA) of *Lychnophora* species is shown in in the Supporting Material (Figure D in S1 File).

Most species belonged to groups (4B) and (4C), whereas only *E*. *capitatus* (Eca) and *E*. *polycephalus* (Epo) belonged to group (4A). On the other hand, *E*. *pabstii* (Epa) was unique species classified as *Piptolepis*, and it was the only species belonging to group (4D). In addition to species that were classified as *Eremanthus*, *E*. *leucodendron* (Ele), classified as Bahian *Lychnophora* and *E*. *crotonoides* (Ecr), were part of group (4B).

### Discriminant variables

In accordance with the groups obtained in PCA and HCA, discriminant variables were assessed by orthogonal partial least square discriminant analysis (OPLS-DA) and a variable importance plot (VIP) provided variables important for discrimination of each class. Then, discriminant compounds were identified for each formed group in multivariate analysis.

Concerning discriminant compounds provided by OPLS-DA for formed groups in principal component analysis (PCA) of 78 species (Fig 1), flavonoids and sesquiterpene lactones were important for segregation of these species since they were found as discriminants in the four groups; those that were able to discriminate each group were identified. Table 2 presents the compound identities and the annotated compounds differentially accumulating in each group. Detailed information for identification of these compounds is provided as Supporting Material. In addition, the chemical structures of these compounds are represented in Figure E in S1 File.

**Table 2:** Identification of discriminant compounds of each group obtained in OPLS-DA of 78 species from the Lychnophorinae subtribe and data taken from UHPLC-MS and UHPLC-HCD MS/MS analyses.

Compound	Rt (min)^a^	Positive Ionization TIC Chromatogram Ions (*m/z*)	HCD MS/MS in positive ionization	Negative Ionization TIC Chromatogram Ions (*m/z*)	HCD MS/MS in negative ionization	UV max (nm)	Groups^b^
vicenin-2 ^c^	8.4	[M + H]^+^ 595.17 bp,	595→ 577, 559, 541, 523, 481, 457 bp, 439, 409, 379, 355, 337, 325, 295	[M − H]^-^ 593,15 bp	593→ 473, 383, 353	271, 334	1A, 4B
kaempferol	13.3	[M + H]^+^ 287.06 bp	287	[M − H]^-^ 285.04 bp	285→ 165	252, 268 sh, 344	1A
3′,4′,7-tri-*O*-methylquercetin	17.0	[M + H]^+^ 345.10 bp, 316.32	345→ 330 bp, 316, 287, 259, 231	[M − H]^-^ 343.08 bp	343→ 328, 313 bp, 301, 285, 270	254, 280 sh, 348	1B
orientin	7.7	[M + H]^+^ 449.12 bp, 369.12	449→ 431, 413, 383, 353, 329 bp, 299	[M − H]^-^ 447.63.11 bp	447→ 357, 327	255, 266, 346	1C
2′′-coumaroylisoorientin	11.6	[M + H]^+^ 595.14 bp	595→ 317, 287 bp	[M − H]^-^ 593.13, 543.15	593→ 447	267, 290 sh, 314	
15-hydroxyeremantholide B	13.6	[M + H]^+^ 379.17 bp, [(M + H)—H_2_O]^+^ 361.16	379→ 361 bp, 317, 293, 271, 259, 231, 203	[M − H]^-^ 377.16 bp	377→ 315, 257, 239	268	
6-hydroxyluteolin-*O*-dipentoside	9.3	[M +Na]^+^ 589.15, 295.08, 277.07 bp,	589→ 287, 163 bp	[M − H]^-^ 565.16	565→ 179	297, 326	1D
5-hydroxy-7,3,4-trimetoxyflavone	22.5	[M + H]^+^ 329.10 bp	329→ 314, 299, 271, 243		-	254, 280 sh, 345	
isorhamnetin-3-*O*-glucoside	9.4	[M + H]^+^ 479.12 bp, [(M + H) -162]^+^ 317.07	479→ 317, 163 bp	[M − H]^-^ 477.10 bp	477→ 315, 314, 289, 285, 271, 243	253, 341	3A
4′-*O*-methyleriodictyol	13.0	[M + H]^+^ 303.09. bp	303→ 289, 257, 153 bp	[M − H]^-^ 301.07 bp	301→ 287	288, 323sh	
3-*O*-methylquercetin ^c^	14.2	[M + H]^+^ 317.07 bp	317→ 302 bp	[M − H]^-^ 315.05 bp	315→ 300 bp, 271, 255	255, 266, 313	3B, 4D
15-hydroxy-16α-(1′-methylprop-1′-Z-enyl)-eremantholide ^c^	16.2	797.27, 775.29 [M + Na]^+^ 399.14, [M + H]+ 377.12, [M + H—(H2O)]+ 359.15, 333.13 bp	797→ 399 bp, 359, 333, 315	-	-	267	3B
pinocembrin	18.2	[M + H]^+^ 257.08 bp	257→ 153 bp	[M − H]^-^ 255.07 bp	255→ 213, 211 bp, 171	289	
3-*O*-acetylpinobanksin	18.1	[M + H]^+^ 315.09	315→ 273, 255, 227 bp, 199, 181, 153	[M − H]^-^ 313.07	313→ 271, 253 bp, 225, 197	293, 345	
15-acetoxygoyazensolide	14.2	743.23, 721.25 [M +H]+ 361.13 bp,	361, 343, 291, 257, 229 bp, 211, 183, 163				3C
15-desoxygoyazensolide	18.0	711.24, 689.26 [M +H]+ 345.14 bp, [M +Na]+ 367.11	367, 345, 259, 231, 213, 185 bp	[M − H]^-^ 343.13, 329.07 bp, 299.06, 277.07, 215.13		268	3C, 4C
7-*O*-methyl-apigenin	18.9	[M +H]+ 285.14 bp	285→ 215bp	[M − H]^-^ 283.06 bp	283→ 269, 239, 211 bp	265, 330	3C
quercetin-3-*O*-(caffeoyl)-glucoside	9.8	[M + H]^+^ 627.13	627→ 303, 163 bp	[M − H]^-^ 625.12 bp, 515.12, 487.13	625→ 463, 301	252, 290 sh, 332	3D
kaempferol-3-*O*-rutinoside	11.3	[M + H]^+^ 595.15 bp	595→ 287 bp, 177	[M − H]^-^ 593.13	593→ 285 bp	267, 314	
hexahydroxy-4-guaien-12,6-olide	14.7	[M + H]^+^ 331.08 bp		[M − H]^-^ 329.07 bp	329→ 314, 299 bp, 271	254	
luteolin-6,8-di-*C*-hexoside	6.2	[M + H]^+^ 611.16 bp	611→ 593, 575, 557, 539, 497, 473 bp, 455, 425, 395, 371, 353, 341, 311, 277, 255	[M − H]^-^ 609.15 bp	609→ 489, 471, 429, 399, 369, 191, 161	272, 280 sh, 336	4A
quercetin-3-*O*-(4″′-*O*-*trans*-caffeoyl)-α-rhamnopyranosyl-(1→6)-β-galactopyranoside ^c^	7.1	[M + H]^+^ 773.19 bp	773→755, 737, 707, 677, 653, 635, 627, 587, 557, 533, 503, 471, 437, 395, 353, 325, 303, 277, 255, 163	[M − H]^-^ 771.16 bp	771→ 609, 591, 531, 471, 369	273, 280 sh, 335	
kaempferol-3-*O*-hexose-*O*-caffeoyl-*O*-rhamnoside	8.6	[M + H]^+^ 757.20	757→739, 637, 611, 577, 541, 517, 471, 457, 439, 379, 355, 337, 325, 287, 177, 163	[M − H]^-^ 755.18 bp, 652.18, 579.17	755→ 593, 575, 473, 455, 335, 179	272, 300, 330	
luteolin	10.9	[M + H]^+^ 287.06 bp	287	[M − H]^-^ 285.04 bp	285→ 199	253, 262 sh, 293 sh, 347	
apigenin	12.2	[M + H]^+^ 271.06 bp	271→229, 153	[M − H]^-^ 269.05 bp	269	268, 333	
kaempferol	12.4	[M + H]^+^ 287.06 bp	271→165	571.09, [M − H]^-^ 285.04 bp	285	265, 289, 365	
isoorientin-3”-*O*-glucopyranoside	13.1	[M + H]^+^ 611.12 bp, 549.23	611→ 287, 163 bp	[M − H]^-^ 609.12 bp, 547.22	609→ 547, 323, 285, 179, 161	264, 288 sh, 329	4C
3-*O*-methylkaempferol	15.6	[M + H]^+^ 301.07 bp	301→286, 258 bp, 229, 165	[M − H]^-^ 299.06 bp	299→ 284 bp, 256, 227	267, 290, 346	
eremantholide A	19.0	[M + H]^+^ 349.16 bp, [M + H—(H2O)]+ 331.15	349→283, 268 bp, 239, 211	-	-	268	
ermanin	19.5	[M + H]^+^ 315.09 bp	315→ 300 bp, 285, 257, 229, 201	[M − H]^-^ 313.07 bp	313→ 298, 283, 255, 211, 183	267, 280 sh, 340	
not identified	15.2	[M + Na]^+^ 395.29 bp, [M + H]+ 373.62	395→373, 361, 327, 267, 255 bp			246	4D

^a^ Retention time

^b^Groups formed by 78 species in PCA and HCA

^c^Identification based on authentic standards

Among species belonging to group (1A), vicenin-2 and kaempferol were identified, while 6-hydroxyluteolin-*O*-dipentoside and 5-hydroxy-7,3,4-trimetoxyflavone were found for species of group (1D). In addition, 3′,4′,7-tri-*O*-methyl quercetin was identified in group (1B), while orientin, 2′′-coumaroylisoorientin and 15-hydroxyeremantholide B were found in group (1C).

Regarding discriminant compounds for formed groups under PCA of *Lychnophora* (Fig 3), which was analyzed separately, the identity of almost all SLs varied among the different groups, which allowed discrimination. Species belonging to group (3B) exhibited 15-hydroxy-16α-(1′-methylprop-1′-*Z*-enyl)-eremantholide, while group (3C) exhibited 15-acetoxygoyazensolide and 15-desoxygoyazensolide. On the other hand, group (3D) exhibited hexahydroxy-4-guaien-12,6-olide.

In addition to the SLs, flavonoids were also present with great variety among the different groups of *Lychnophora*. Group (3A) exhibited isorhamnetin-3-*O*-glucoside and 4′-*O-*methyleriodictyol, while group (3B) exhibited 3-*O*-methylquercetin, 3-*O*-acetylpinobanksin and pinocembrin. Compound 7-*O*-methyl-apigenin was only found in group (3C) and group (3D) exhibited quercetin-3-*O*-(caffeoyl)-glucoside and kaempferol-3-*O*-rutinoside. These compounds are represented in Figure F in S1 File.

Concerning discriminant compounds for formed groups under PCA of *Eremanthus* (Fig 4), flavonoids were also important for discriminating all groups of *Eremanthus* species because they displayed great variety among the different groups. Species belonging to group (4A) exhibited luteolin-6,8-di-*C*-hexoside, quercetin-3-*O*-(4″′-*O*-*trans*-caffeoyl)-α-rhamnopyranosyl-(1→6)-β-galactopyranoside, kaempferol-3-*O*-hexose-*O*-caffeoyl-*O*-rhamnoside, luteolin, apigenin and kaempferol, while isorhamnetin-3-*O*-glucoside, isoorientin-3”-*O*-glucopyranoside and vicenin-2 were exhibited in species from group (4B). Species belonging to group (4C) exhibited ermanin and 3-*O*-methylkaempferol, whereas group (4D), represented by *E*. *pabstii* (Epa) species, exhibited 3-*O*-methylquercetin. Regarding SLs, it should be noted that they discriminated species belonging to groups (4C) and (4D). Therefore, group (4C) exhibited eremantholide A and 15-desoxygoyazensolide, whereas *E*. *pabstii* (Epa) from group (4D) exhibited an unidentified SL. These compounds are represented in Figure G in S1 File.

These results showed better congruence with the phylogenetic hypothesis when the metabolomic data were restricted to a single genus (*Lychnophora* or *Eremanthus*), whereas the metabolomic data seemed to be rather incongruent when all Lychnophorinae were taken into account. This noisy pattern at a higher taxonomic level probably reflects the existence of biological or physiological processes, such as convergence, adaptation or metabolite quantitative fluctuations that may obscure the phylogenetic signal. This is noteworthy on account of the harsh environmental conditions that prevail along the *Campo rupestre* and *Cerrado* ecosystems, including extreme oligotrophic and acid substrates, constant wind exposure, and intense fire regime [26]. Further studies are necessary for a better understanding of the processes leading to similar metabolic profiles in species not closely related. Here, in the same way that was done by Loeuille et al. (2015b), only one specimen of each species was analyzed for the purpose of mutual comparison. This could also explain the noisy pattern at higher taxonomic level. It is worthy note that most of the species studied are micro-endemics presenting very few populations; such a scenario offers a low probability for infraspecific chemical qualitative variation.

Notably, certain care must be taken when comparing multivariate analyses results with a phylogenetic hypothesis. The latter analysis is based on special similarity (homology, similarity inherited from a common ancestor) whereas multivariate analyses use an overall or global similarity without distinction between homology and homoplasy (the latter does not reflect evolutionary relationship) [27]. The incongruence noted with a phylogenetic hypothesis when all Lychnophorinae were taken into account may be attributed to such methodological intrinsic differences and the robustness of metabolomic analysis combining both UHPLC-MS and GC-MS data, which provided a more widespread metabolomics approach.

When considering the two most speciose genera, their similarity became even more evident. The *Lychnophora* species (Fig 3) classified as *Lychnophora* by Loeuille et al. (2015b) clustered almost all together into group (3C), as well as species classified as *Prestelia* Alliance, which clustered mainly into the close groups (3D) and (3C). In addition, all species classified as Bahian *Lychnophora* remained clustered into the same group (3B).

Regarding *Eremanthus* (Fig 4), species classified in this genus clustered almost all together into group (4C). The remaining species clustered into group (4A) and mainly into group (4B), with *E*. *crotonoides* and *E*. *leucodendron*, the unique species of that genus classified as Bahian *Lychnophora* [5]. In addition, *E*. *pabstii* was segregated into group (4D) according to its classification as the unique species of that genus *Piptolepis* [5].

## Conclusions

We have recently reported the use of a similar approach on a small scale in a study of the genus *Vernonia* Schreb [22]. In this research, a preliminary study investigating the possibilities of using metabolomics for chemotaxonomical purposes was performed with a restricted group of ten species. In the present study, we not only greatly expanded the number of sampled species but also used a much more comprehensive raw data set for the statistical analyses, which included GC-MS and UHPLC-MS (both positive and negative ionization modes) combined. Our results using these improvements seemed to confirm that the metabolomics approach might be a promising tool for chemotaxonomical studies with taxonomic purposes but also highlighted the importance of choosing the correct taxonomic level; otherwise, the phylogenetic signal might be obscured by biological or physiological processes that may play a major role in the evolution of lineages on harsh habitats. The use of metabolomics as the primary data source for phylogenetic inference, in addition to molecular and morphological data, would offer an opportunity to obtain a robust phylogenetic hypothesis for the genera of Lychnophorinae subtribe and insight into the evolution of the secondary metabolites of this group.

## Supporting information

S1 File (DOCX)
