# Supplementary material for: Comprehensive untargeted metabolomics of Lychnnophorinae subtribe (Asteraceae: Vernonieae) in a phylogenetic context
Source: PLoS One. 2018 Jan 11;13(1):e0190104. doi: 10.1371/journal.pone.0190104 (PMC5764248; doi:10.1371/journal.pone.0190104)
Supplement: S1 File — (DOCX) [file pone.0190104.s001.docx]

## Supporting Material

Article title: Comprehensive untargeted metabolomics of Lychnnophorinae subtribe (Asteraceae: Vernonieae) in a phylogenetic context

*Authors: Maria Elvira Poleti Martucci, Benoit Loeuille, José Rubens Pirani, Leonardo Gobbo-Neto.*

**Plant Material**

Voucher materials were mainly deposited in herbarium SPF and some indicated vouchers were deposited in herbariums UEC, HUEFS and HUFU. Theses vouchers were deposited under the codes Loeuille et al. 441 (*Anteremanthus hatschbachii* H. Rob., collected at Grão Mogol - Aha), Loeuille et al. 394 (*Blanchetia heterotricha* DC., collected at Morro do Chapéu - Bhe), Loeuille and Albergaria Pena 460 (*Chronopappus bifrons* (DC. ex Pers.) DC., collected at Catas Altas - Cbi), Loeuille et al. 510 (*Eremanthus arboreus* (Gardner) MacLeish, collected at Crato - Ear), Loeuille et al. 839 (*E. argenteus* MacLeish & H. Schumach., collected at Alto Paraíso - Earg), Loeuille et al. 836 (*E. auriculatus* MacLeish & H. Schumach., collected at Alto Paraíso - Eau), Loeuille et al. 71 (*E. brevifolius* Loeuille, collected at Congonhas do Norte - Ebr), Loeuille et al. 313 (*E. capitatus* (Spreng.) MacLeish, collected at Abaíra - Eca), Loeuille et al. 306 (*E. cinctus* Baker, collected at Uberlândia - Eci ), Loeuille et al. 25 (*E. crotonoides* (DC.) Sch.Bip., collected at Santana do Riacho – Ecr), Loeuille et al. 430 (*E. elaeagnus* (Mart. ex DC.) Sch.Bip., collected at Joaquim Felicio - Eel), Loeuille et al. 51 (*E. erythropappus* (DC.) MacLeish, collected at Capitólio - Eer), Loeuille et al. 438 (*E. glomerulatus* Less., collected at Francisco Sá - Egl), Loeuille et al. 295 (*E. goyazensis* (Gardner) Sch.Bip., collected at Pirenópolis – Ego), Loeuille et al. 344 (*E. incanus* (Less.) Less., collected at Abaíra - Ein), Loeuille et al. 347 (*E. leucodendron* Mattf.: Brazil, collected at Abaíra - Ele), Loeuille et al. 457 (*E. mattogrossensis* Kuntze, collected at Diamantina - Ema), Loeuille et al. 305 (*E. mollis* Sch.Bip., collected at Pirenópolis - Emo), Loeuille et al. 833 (*E. pabstii* G.M. Barroso, collected at Cristalina - Epa), Loeuille et al. 442 (*E. polycephalus* (DC.) MacLeish, collected at Grão Mogol - Epo), Loeuille et al. 419 (*Eremanthus* sp. X *Paralychnophora reflexoauriculata* (G.M. Barroso) MacLeish, collected at Jacobina - EXPr), Loeuille et al. 840 (*E. uniflorus* MacLeish & H. Schumach., collected at Alto Paraíso - Eun), Loeuille et al. 837 (*E. veadeiroensis* H. Rob., collected at Alto Paraíso - Eve), Loeuille et al.821 (*Gorceixia decurrens* Baker, collected at Leme do Prado - Gde), Loeuille et al. 450 (*Heterocoma albida* (DC. ex Pers.)DC., collected at Serro - Hal), Loeuille et al. 838 (*H. ekmanianum* (Philipson) J.R. Grant, collected at Alto Paraíso - Hek), Loeuille et al. 520 (*H. gracilis* Loeuille, J.N. Nakaj. & Semir, collected at São Gonçalo do Rio Preto - Hgr), Loeuille et al. 635 (*H. lanuginosa* (Glaz. ex Oliv.) Loeuille, J.N. Nakaj. & Semir, collected at Serro - Hla), Loeuille et al. 266 (*H. robinsoniana* Loeuille, J.N. Nakaj. & Semir, collected at São Roque de Minas - Hro), Loeuille et al. 259 (*Hololepis pedunculata* (DC. ex Pers.) DC., collected at São Roque de Minas - Hop), Oliveira 43 - HUEFS (*Lychnophora bishopii* H. Rob., collected at Mucugê - Lbi), Harley et al. PCD 4427 - HUEFS (*L. crispa* Mattf., collected at Rio de Contas - Lcr), Loeuille et al. 108 (*L. diamantinana* Coile & S.B. Jones, collected at Diamantina - Ldi), Loeuille et al. 834 (*L. ericoides* Mart., collected at Alto Paraíso - Ler), Loeuille et al. 67 (*L. gardneri* Sch.Bip., collected at Congonhas do Norte - Lga), Loeuille et al. 445 (*L. granmogolensis* (Duarte) Semir in D.J.N. Hind, collected at Cristália - Lgr), Zappi et al. 2325 (*L. humillima* Sch.Bip., collected at Santana do Pirapama - Lhu), Mello-Silva et al. 3186 (*L.*“*itacambirensis*” sp. ined., collected at Itacambira - Lit), Loeuille et al. 112 (*L.*“*joliana*” sp. ined, Santana do Riacho - Ljo), Loeuille et al. 440 (*L. markgravii* G.M. Barroso, collected at Grão Mogol - Lma), Loeuille et al. 507 (*L. mellobarretoi* G.M. Barroso, collected at Santana do Riacho - Lme), Mello-Silva et al. 463 (*L. “mellosilvae”* sp. ined, collected at Mato Verde - Lmel), Loeuille et al. 337 (*L. passerina* (Mart. ex DC.) Gardner, collected at Abaira - Lpa), Oliveira et al. 471 (*L. pinaster* Mart., collected at Catas Altas - Lpi), Loeuille et al. 448 (*L. ramosissima* Gardner, collected at Josenópolis - Lra), Loeuille et al. 346 (*L. regis* H. Rob., collected at Abaira - Lre), Loeuille et al. 841 (*L. salicifolia* Mart., collected at Alto Paraíso - Lsa), Ferreira et al. 268 - HUEFS (*L. santosii* H. Rob., collected at Mucugê -Lst), Loeuille et al. 79 (*L. sellowii* Sch.Bip., collected at Congonhas do Norte -Lse), Loeuille et al. 483a (*L. syncephala* (Sch.Bip.) Sch.Bip., collected at Santana do Riacho - Lsy), Loeuille et al. 93 (*L. tomentosa* (Mart. ex DC.) Sch.Bip., collected at Diamantina - Lto), Loeuille et al. 333 (*L. triflora* (Mattf.) H. Rob., collected at Abaíra -Ltr). Loeuille et al. 81 (*L. villosissima* Mart., collected at Congonhas do Norte - Lvi), Loeuille et al. 435 (*Lychnophoriopsis candelabrum* (Sch.Bip.) H. Rob., collected at Buenópolis - Lpca), Loeuille et al. 77 (*L. damazioi* (P. Beauv.) H. Rob., collected at Congonhas do Norte - Lpda), Rosa et al. 935 - HUFU (*L. hatschbachii* H. Rob., collected at Diamantina -Lpha), Nakajima et al. 4624 (*Minasia alpestris* (Gardner) H. Rob., collected at Diamantina - Mal), Loeuille et al. 433 (*M. cabralensis* H. Rob, collected at Joaquim Felício - Mca), Loeuille et al. 494 (*M. “cipoensis*” sp. ined, collected at Santana do Riacho - Mci), Loeuille et al. 97 (*M. scapigera* H. Rob., collected at Diamantina - Msc), Loeuille et al. 432 (*M. ramosa* Loeuille, H. Rob. & Semir, collected at Joaquim Felício - Mra), Ferreira et al. 267 - HUEFS (*Paralychnophora atkinsiae* D.J.N. Hind, collected at Mucugê - Pat), Loeuille et al. 330 (*P. bicolor* (DC.) MacLeish, collected at Abaira - Pbi), Loeuille et al. 451 (*P. glaziouana* Loeuille, collected at Serro - Pgl), Loeuille et al. 309 (*P.harleyi* (H. Rob.) D.J.N. Hind, collected at Piatã - Pha), Loeuille et al. 329 (*P. patriciana* D.J.N. Hind, collected at Abaira - Ppa), Loeuille et al. 326 (*P.patriciana* D.J.N. Hind X *P. bicolor* (H. Rob.) D.J.N. Hind, collected at Abaira - PpXPb), Loeuille et al. 396 (*P. reflexoauriculata* (G.M. Barroso) MacLeish, collected at Morro do Chapéu - Pre), Loeuille et al. 17 (*Piptolepis ericoides* Sch.Bip., collected at Santana do Riacho - Pie), Loeuille et al. 517 (*P.oleaster* (Mart. ex DC.) Sch.Bip., collected at São Gonçalo do Rio Preto - Pio), Loeuille et al. 564 (*P. monticola* Loeuille , collected at Santo Antonio do Itambé - Pim), Loeuille et al. 516 (*P.*“*riparia*” sp. ined., collected at São Gonçalo do Rio Preto - Pir), Loeuille et al. 72 (*P. schultziana* Loeuille& D.J.N. Hind, collected at Congonhas do Norte - Pis), Loeuille et al. 113 (*Prestelia eriopus* Sch.Bip., collected at Santana do Riacho - Prer), Mansanares and Verola 340 - UEC (*Prestelia*“*robusta*” sp. ined, collected at Diamantina - Prro), Loeuille et al. 344 (*Proteopsis argentea* Mart. &Zucc. ex DC., collected at Grão Mogol - Prar), Mello-Silva et al. 3008 (*P.*“*hermogenesii*” sp. ined., collected at Botumirim - Prhe).

**Compounds identification**

Luteolin and kaempferol were identified by UV spectrum and both showed the same precursor ion at *m/z* 287 [M + H]^+^. In the other hand, the product ions formed in HCD mass spectra allowed distinction between them. Then, luteolin showed a characteristic ion at *m/z* 199 [1] during HCD mass spectrum obtained in negative ionization mode, whereas, kaempferol had *m/z* 165 as characteristic product ion [2].

Apigenin showed UV maxima at 268 and 333 nm [3]. The mass spectrum obtained in negative ionization mode showed a precursor ion at *m/z* 269 [M - H]^-^ and the mass spectrum obtained in positive ionization mode showed a precursor ion at *m/z* 271 [M + H]^+^. Also, HCD mass spectrum obtained in positive ionization mode showed a characteristic ion at *m/z* 153.

Pinocembrin showed precursor ions at *m/z* 257 [M + H]^+^ and *m/z* 255 [M - H]^-^ obtained in positive ionization mode and negative ionization mode, respectively. The HCD mass spectrum obtained in negative ionization mode showed expected fragmentation patterns for this compound [4].

Vicenin-2 (6,8-di-*C*-*β*-glucupyranosylapigenin) showed UV maxima at ≈ 271 and 334 nm and precursor ions and *m/z* 593 [M - H]^-^ and *m/z* 595 [M + H]^+^ [2,5,6]. The fragmentation pattern confirmed this identification, then HCD mass spectrum in the negative ionization mode showed product ions at *m/z* 473, *m/z* 383 and *m/z* 353. This compound was compared with authentic standard [5].

The compound luteolin-6,8-di-*C*-hexoside showed a precursor ion at *m/z* 609 [M - H]^-^ and its HCD mass spectrum presented product ions with a characteristic fragmentation pattern of 6,8-di-C-hexosyl flavones. At the negative ionization mode, it is possible to note the product ion at *m/z* 399, which represents the aglycone plus the residue of the sugars linked to it and therefore indicates the aglycone as trihydroxiflavone (luteolin, 286*u*) [7].

Orientin (luteolin-8-*C*-glycoside) was identified by UV spectrum characteristic for a 3´,4´-diOH system in flavones. Also HCD mass spectrum of precursor ion at *m/z* 447 [M - H]^-^ showed product ions at *m/z* 357 and *m/z* 327. Both ions indicate the presence of mono-C-glycosides. In addition, it is important to note that the absence of a fragment ion at *m/z* 429 is characteristic of orientin [7].

The compound 6-hydroxyluteolin-*O*-dipentoside showed UV spectrum characteristic for luteolin. The mass spectra showed precursor ions at *m/z* 589 [M + Na]^+^ and *m/z* 565 [M - H]^-^. Also the HCD mass spectrum obtained in positive ionization mode showed an ion at m/z 287, correspondent to aglycone luteolin [7].

Isoorientin-3”-*O*-glucupyranoside showed UV spectrum with maxima absorptions at 264 and 329 nm and a shoulder at 288 nm. Also this compound showed precursor ions at *m/z* 609 [M - H]^-^ and at *m/z* 611 [M + H]^+^ [8].

Kaempferol-3-*O*-hexose-caffeoyl-rhamnoside showed precursor ions at *m/z* 757 [M + H]^+^ and *m/z* 755 [M - H]^-^. The HCD mass spectrum obtained in the positive ionization mode showed product ions at *m/z* 611, formed after loss of a rhamnosyl unit, *m/z* 471, *m/z* 325, *m/z* 287 and *m/z* 163 [9].

The compound kaempferol-*3-O*-rutinoside showed a precursor ion at *m/z* 593 [M - H]^-^ and HCD mass spectrum obtained in negative ionization mode presented a product ion at *m/z* 285 attributed to the elimination of a rutinoside residue. Also in the positive ionization mode, HCD mass spectrum showed an ion at *m/z* 287 formed after elimination of rutinoside residue [9].

Quercetin-3-*O*-(4″′-*O*-*trans*-caffeoyl)-α-rhamnopyranosyl-(1→6)-β-galactopyranoside showed a precursor ion at *m/z* 773 [M + H]^+^ in the positive ionization mode. It is important to note that HCD mass spectrum obtained in the positive ionization mode showed product ions at *m/z* 627, *m/z* 471, *m/z* 325 and *m/z* 303, which is product ion characteristics for quercetin [10]. This compound was compared with authentic standard [9]

The compound 3-*O*-acetylpinobanksin showed UV spectrum typical for pinobanksin. Also this compound showed precursor ions at *m/z* 315 [M + H]^+^ and *m/z* 313 [M - H]^-^ . The HCD mass spectrum obtained in the negative ionization mode showed product ions at *m/z* 253 and *m/z* 271 formed after neutral losses of acetic acid and C_2_H_2_O, respectively. Whereas the HCD mass spectrum obtained in positive ionization mode showed product ions at *m/z* 153, due to substituents in A ring of this flavonoid [4].

The compound 3-*O*-methylquercetin showed UV spectrum typical for flavones. In addition, this compound showed precursor ions at *m/z* 317 [M + H]^+^ and *m/z* 315 [M - H]^-^. The HCD mass spectrum obtained in both ionization modes showed loss of methyl group [10]. This compound was compared with authentic standard [4]

The compound 3',​4',​7-​tri-*O*-methylquercetin showed UV spectrum typical for flavones. The mass spectra showed precursor ions at *m/z* 345 [M + H]^+^ and *m/z* 343 [M - H]^-^. Also HCD mass spectrum obtained in positive ionization mode showed an ion at *m/z* 330 as base peak, formed after loss of two methyl groups. Whereas, HCD mass spectrum obtained in negative ionization mode showed an ion at *m/z* 301, characteristic for quercetin [2].

The compound 5-hydroxy-7,3,4-trimethoxyflavone [11] showed UV maxima at 254 and 345 nm and a shoulder at 280 nm. The mass spectra showed precursor ions at *m/z* 329 [M + H]^+^ and *m/z* 327 [M - H]^-^. In addition, HCD mass spectrum obtained in positive ionization mode showed ions at *m/z* 314 and *m/z* 299 formed after loss of one and two methyl groups, respectively.

The compound 3-*O*-methylkaempferol showed UV spectrum similar to that obtained for kaempferol. Also the precursor ions at *m/z* 303 [M + H]^+^ and *m/z* 301 [M - H]^-^ and HCD mass spectra obtained in both ionization modes were characteristic for this compound, mainly ion at *m/z* 165 in the positive ionization mode [12].

The compound 4′-methyleryodictyol showed UV maxima at 288 nm and a shoulder at 323 nm. The precursor ions obtained in positive and negative ionization modes were *m/z* 303 [M + H]^+^ and *m/z* 301 [M - H]^-^, respectively. In addition, HCD mass spectra obtained in positive and negative ionization modes showed ions at *m/z* 289 and *m/z* 287, respectively, and both are correspondent to aglycone eryodictyol, formed after loss of a methyl group [1].

The compound 7-*O*-methylapigenin showed UV spectrum similar to that obtained for apigenin. The mass spectra showed precursor ions at *m/z* 285 [M + H]^+^ and *m/z* 283 [M - H]^-^. In addition, HCD mass spectra obtained in positive and negative ionization modes showed ions at *m/z* 271 and *m/z* 269, respectively. Both ions are correspondent to aglycone and were formed after loss of a methyl group [3,9].

The compound isorhamnetin-3-*O*-glucoside [13] was identified due to precursor ions at *m/z* 477 [M - H]^-^ and *m/z* 479 [M + H]^+^. The HCD mass spectrum obtained in positive ionization mode showed ion at *m/z* 317, formed after loss of sugar [14]. Also, HCD mass spectrum obtained in negative ionization mode showed ion at *m/z* 271, which is characteristic for this compound.

The compound 2′′-coumaroylisoorientin showed precursor ions at [M + H]^+^ *m/z* 595 and [M - H]^-^ *m/z* 593. Also, this compound showed UV maxima at 267 and 314 nm and a shoulder at 290 nm. The HCD mass spectrum obtained in negative ionization mode showed ion at *m/z* 447, formed after loss of coumaroyl [8].

The ermanin (5,7-dihydroxy-3’,4’-dimethoxyflavone) [15] showed UV maxima at 267 and 340 nm and a shoulder at 280 nm. The mass spectra showed precursor ions at *m/z* 315 [M + H]^+^ and *m/z* 313 [M - H]^-^. The HCD mass spectrum obtained in positive ionization mode showed ions at *m/z* 300 and *m/z* 285, formed after loss of one and two methyl groups, respectively.

The compound quercetin-3-*O*-(caffeoyl)-glucoside showed UV maxima characterisitic of hydroxycinnamic acid, but fragmentation pattern suggested O-substituted flavonol. The precursor ions were *m/z* 627 [M + H]^+^ and *m/z* 625 [M - H]^-^. In addition, the HCD mass spectrum obtained in negative ionization mode showed ion *m/z* 463, formed after loss of the caffeoyl moiety and the ion *m/z* 301, which is correspondent to aglycone quercetin [16].

The compound 15-hydroxyeremantholide B [15] showed UV maxima at 268 nm, which is typical for eremantholides. The mass spectra showed precursor ions at *m/z* 378 [M - H]^-^ and *m/z* 377 [M + H]^+^. Also the HCD mass spectrum obtained in positive ionization mode showed ions at *m/z* 361, *m/z* 293 and *m/z* 317, which are correspondent to fragmentation pattern of this sesquiterpene lactone.

The compound 15-hydroxy-16α-(1′-methylprop-1′-*Z*-enyl)-eremantholide showed UV maxima at 267 nm. The mass spectrum obtained in positive ionization mode showed precursor ion at *m/z* 377 [M - H]^+^. Also, the HCD mass spectrum obtained in positive ionization mode showed ions at *m/z* 359 and *m/z* 315. This compound was compared with authentic standard [4].

The compound 15-acetoxygoyazensolide showed UV maxima at 268 nm. The mass spectrum obtained in positive ionization mode showed precursor ion at *m/z* 361 [M - H]^+^. Also, the HCD mass spectrum obtained in positive ionization mode showed ions at *m/z* 343, *m/z* 291 and *m/z* 229 [17].

The compound 15-desoxygoyazensolide showed UV maxima at 268 nm and the precursor ion at *m/z* 345 [M – H]^+^. Also the occurrence of this compound in Lychnophorinae subtribe allowed its identification [18,19].

The compound eremantholide A showed UV maxima at 268 nm and a precursor ion at *m/z* 349 [M – H]^+^, obtained in positive ionization mode. Also the literature data and the occurrence of this compound [18,20,21,22].

The compound hexahydroxy-4-guaien-12,6-olide showed UV maxima at 254, typical of guaianolides. Also, this compound was identified by a search in Dictionary of Natural Products.

**References**

1. Sánchez-Rabaneda F, Jauregui O, Lamuela-Raventos RM, Bastida J, Viladomat F, Codina C. Identiﬁcation of phenolic compounds in artichoke waste by high-performance liquid chromatography–tandem mass spectrometry. J Chromatogr A. 2003; 1008: 57-72.
2. Cuyckens F, Claeys M. Mass spectrometry in the structural analysis of ﬂavonoids. J Mass Spectrom. 2004; 39: 1-15.
3. Markham, KR. Techniques of Flavonoid Identification. London; Academic Pres; 1982.
4. Gobbo-Neto L, Lopes NP. Online identification of chlorogenic acids, sesquiterpene lactones, and flavonoids in the Brazilian arnica *Lychnophora ericoides* Mart. (Asteraceae) leaves by HPLC-DAD-MS and HPLC-DAD-MS/MS and a validated HPLC-DAD method for their simultaneous analysis. J Agric Food Chem. 2008; 56: 1193-1204.
5. Gobbo-Neto L, Gates PJ, Lopes NP. Negative ion ‘chip-based’ nanospray tandem mass spectrometry for the analysis of flavonoids in glandular trichomes of *Lychnophora ericoides* Mart. (Asteraceae). Rapid Commun Mass Spectrom. 2008; 22: 3802-3808.
6. Iswaldi I, Arráez-Román D, Rodríguez-MedinaI, Beltrán-Debón R, Joven J,[Segura-Carretero A](http://www.ncbi.nlm.nih.gov/pubmed/?term=Segura-Carretero%20A%5BAuthor%5D&cauthor=true&cauthor_uid=21509483), [Fernández-Gutiérrez A](http://www.ncbi.nlm.nih.gov/pubmed/?term=Fern%C3%A1ndez-Guti%C3%A9rrez%20A%5BAuthor%5D&cauthor=true&cauthor_uid=21509483). Identification of phenolic compounds in aqueous and ethanolic rooibos extracts (*Aspalathus linearis*) by HPLC-ESI-MS (TOF/IT). Anal Bioanal Chem. 2011; 400**:** 3643 – 3654.
7. Breiter T, Laue C, Kressel G, Gröll S, Engelhardt UH, Hahn H. Bioavailability and antioxidant potential of rooibos ﬂavonoids in humans following the consumption of different rooibos formulations. Food Chem. 2011; 128: 338-347.
8. Deng X, Gao G, Zheng S, Li F. Qualitative and quantitative analysis of ﬂavonoids in the leaves of *Isatis indigatica* Fort. by ultra-performance liquid chromatography with PDA and electrospray ionization tandem mass spectrometry detection. J Pharm Biomedical Anal. 2008; 48: 562-567.
9. Martucci MEP, de Vos RCH, Carollo CA, Gobbo-Neto L. Metabolomics as a potential chemotaxonomical tool: application in the genus *Vernonia* Schreb. Plos One. 2014; 9: 1-8.
10. Li J, Jiang H, Shi R. A new acylated quercetin glycoside from the leaves of *Stevia rebaudiana* Bertoni. Nat Prod Res. 2009; 23: 1378-138.
11. Sacilotto ABC, Sartori FT, Vichnewski W. Chemical constituents of *Eremanthus veadeiroensis* (Asteraceae). Biochem Sys Ecol. 2002; 30: 897-900.
12. Bohlmann F, Gupta RK, Jakupovic J, Robinson H, King RM. Three germacranolides and other constituents from *Eremanthus* species. Phytochemistry. 1981; 20: 1609-1612.
13. Grael CFF, Kanashiro A, Kabeya LM, Jordão CO, Takeara R, Gobbo-Neto L, et al. *In vitro* study of antioxidante and scavenger properties of phenolic compound from Lychnophora species. Quim Nova. 2010; 3: 867-870.
14. Santos SAO, Freire CSA, Domingues MRM, Silvestre AJD, Pascoal Neto C. Characterization of phenolic components in polar extracts of *Eucalyptus globules* Labill.Bark by high-performance liquid chromatography mass spectrometry. J Agric Food Chem. 2011; 59: 9386-9393.
15. Vichnewski W, Skrochy CA, Nasi AMTT, Lopes JLC, Herz W. 15-hydroxieremantholide B and derivatives from *Eremanthus arboreus*. Phytochemistry. 1999; 50: 317-320.
16. Francescato LN, Debenedetti SL, Schwanz TG, Bassani VL, Henriques AT. Identification of phenolic compounds in *Equisetum giganteum* by LC-ESI-MS/MS and a new approach to total flavonoid quantification. Talanta. 2013; 105: 192-203.
17. da Costa FB, Dias DA, Lopes JLC, Vichneweski W. Flavonoids and heliangolides from *Lychnophora diamantinana*. Phytochemistry. 1993; 34: 26-263.
18. Bohlmann F, Singh P, Zdero C, Ruhe A, King RM. Robinson H. Furanoheliangolides from two *Eremanthus* species and from *Chresta sphaerocephala*. Phytochemistry. 1982; 21: 1669-1673.
19. Sakamoto HT, Flausino D, Castellano EE, Stark CBW, Gates PJ, Lopes NP. Sesquiterpne lactones from *Lychnophora ericoides*. J Nat Prod. 2003; 66: 693-695.
20. Borella JC, Lopes JLC, Vichnewski W, Cunha WR, Herz W. Sesquiterpene lactones, triterpenes and flavones from *Lychnophora ericoides* and *Lychnophora pseudovillosissima*. Biochem Sys Ecol. 1998; 26: 671-676.
21. Davalian, D, Garrat PJ. Eremantholide A, a novel tumor inhibiting compound from *Eremanthus elaeagnus* Schultz-Bip. (Compositae). J Am Chem Soc, 1975. 97: 6884-6886.
22. Herz W, Kumar N. Cytotoxic sesquiterpene lactones of *Eremanthus incanus* and *Heterocoma albida*. Crystal structures and stereochemistry of eregoyazin. J. Org. Chem. 1980; 45: 2503-2506.

**Supporting Figures**

**
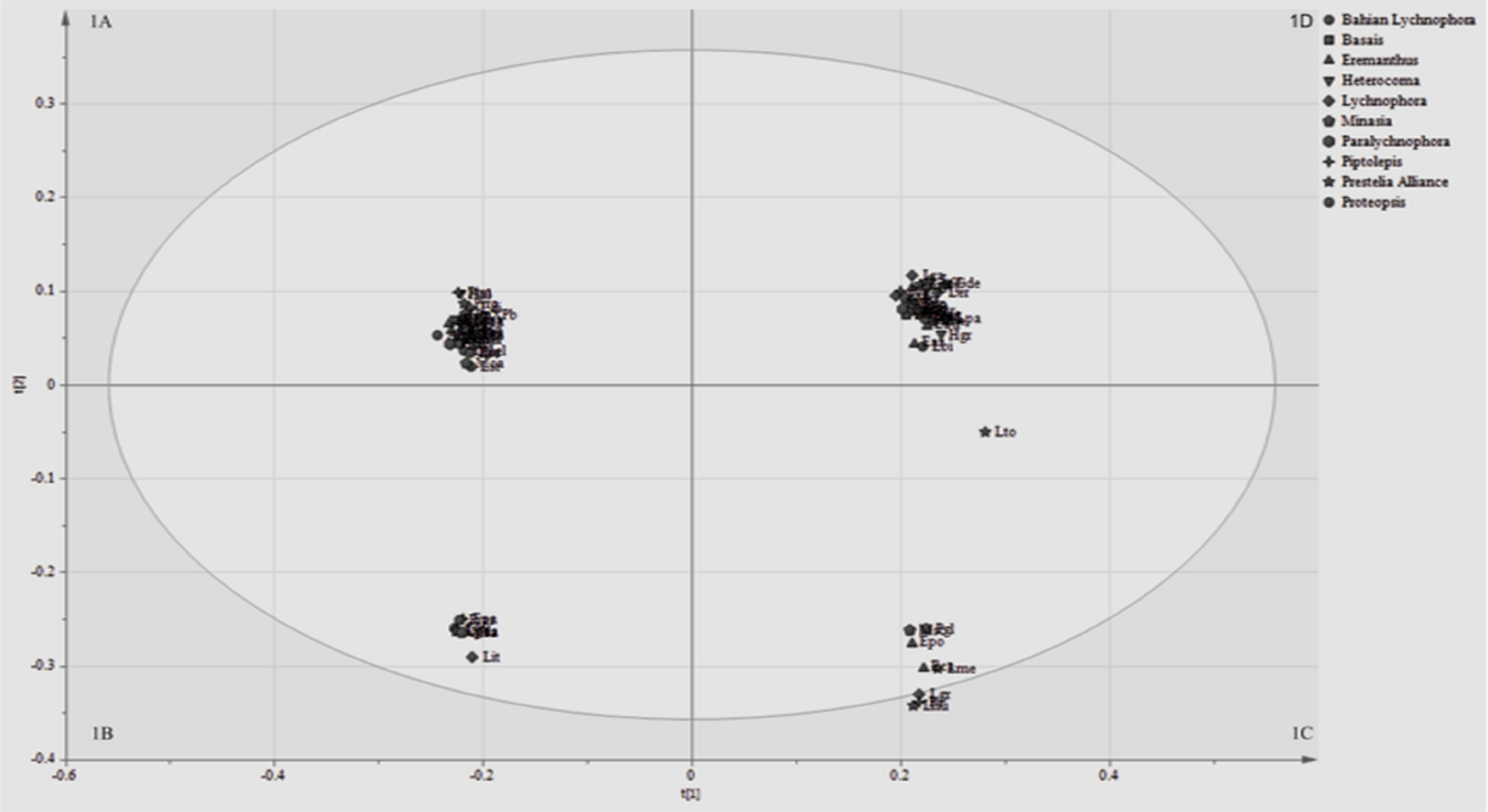
**

**Supporting Fig. S1** Score scatter plots of Principal Component Analysis (t1 versus t2) of 78 analyzed species from Lychnophorinae subtribe. Based on metabolic profiling obtained in LC-MS, in both positive and negative electrospray ionization (ESI) modes.


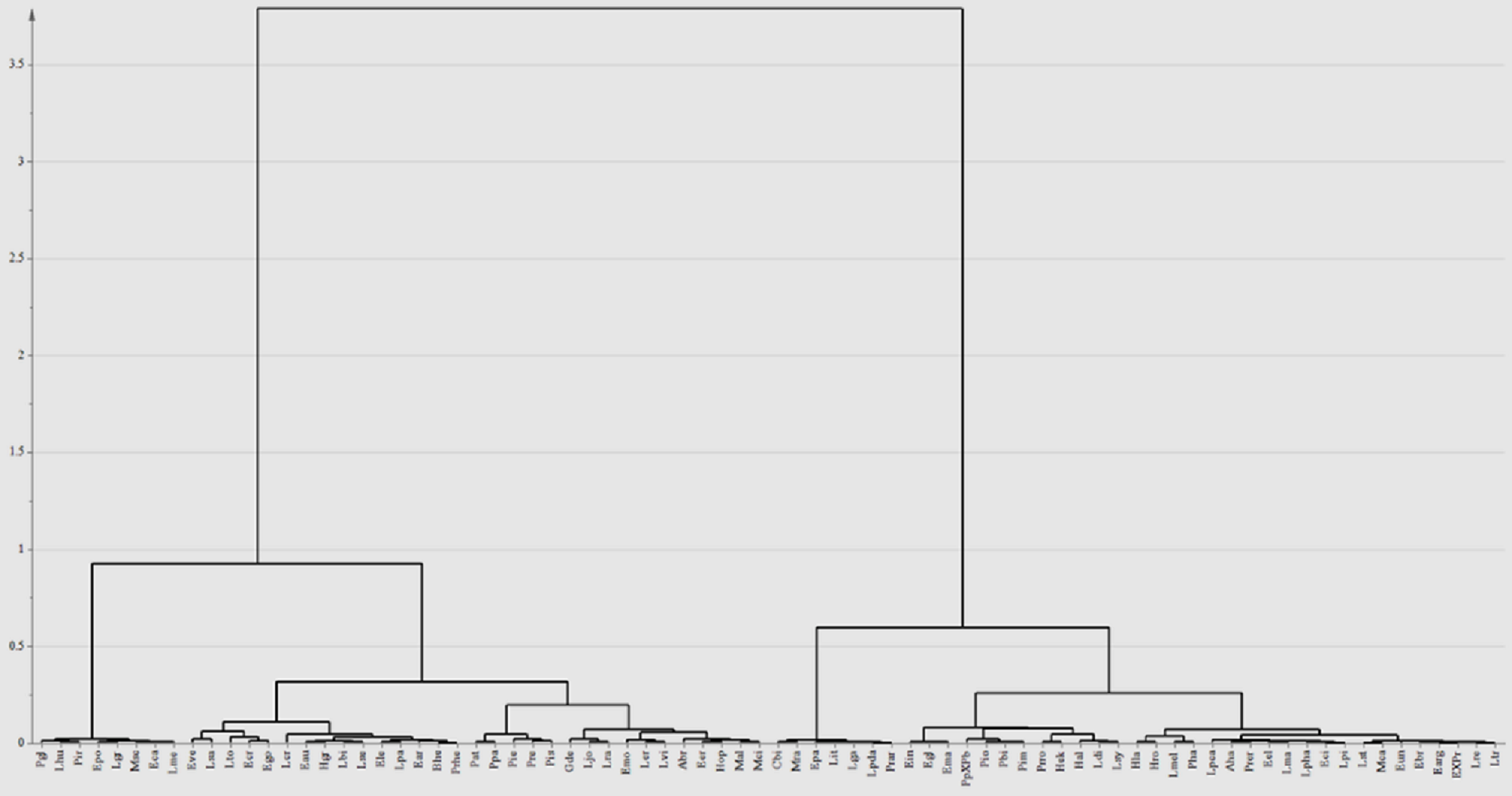


**Supporting Fig. S2** Hierarchical Cluster Analysis of 78 analyzed species from Lychnophorinae subtribe. Based on metabolic profiling obtained in LC-MS, in both positive and negative electrospray ionization (ESI) modes.


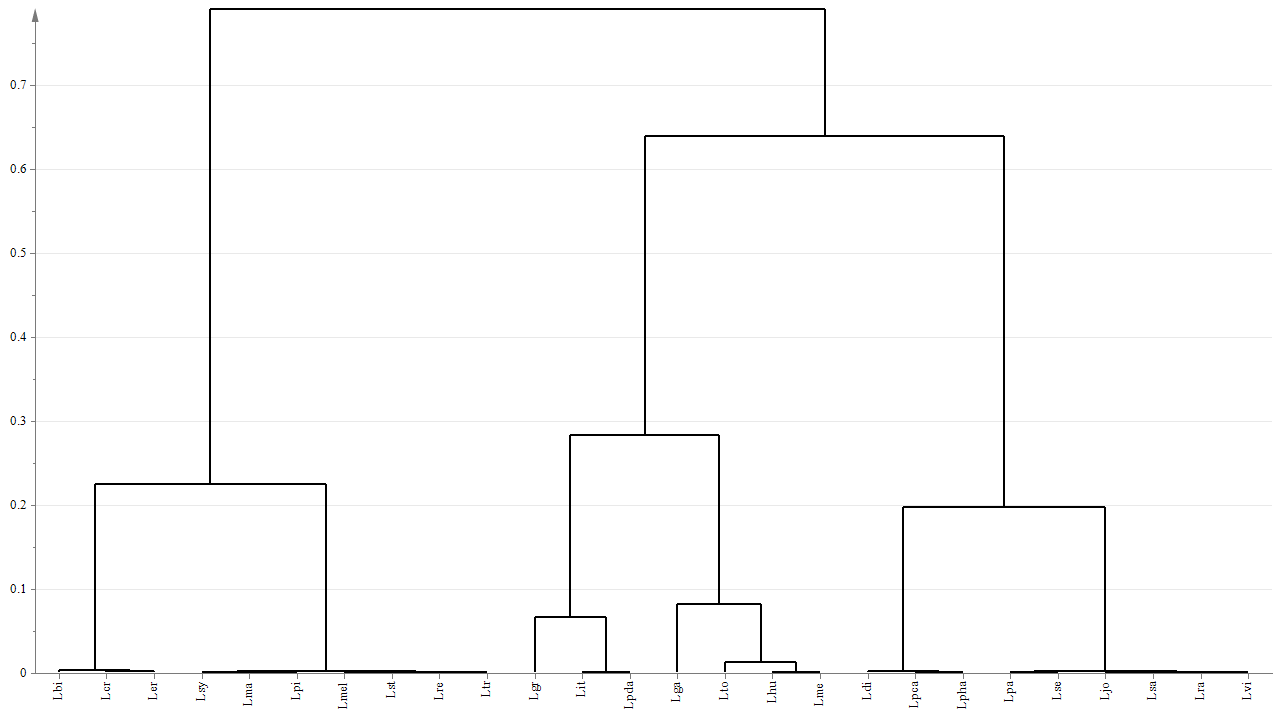


**Supporting Fig. S3** Hierarchical Cluster Analysis of *Lychnophora* species. Based on metabolic profiling obtained in LC-MS, in both positive and negative electrospray ionization (ESI) modes, and in GC-MS.


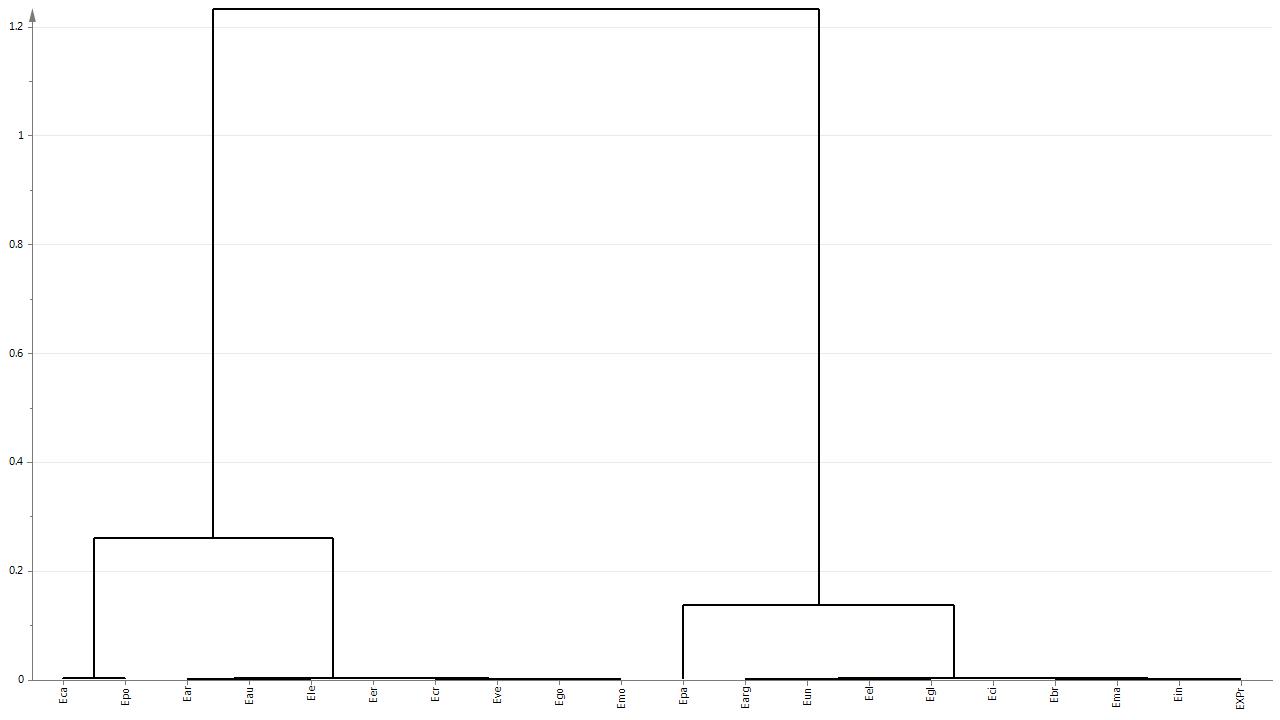


**Supporting Fig. S4** Hierarchical Cluster Analysis of *Eremanthus* species. Based on metabolic profiling obtained in LC-MS, in both positive and negative electrospray ionization (ESI) modes, and in GC-MS.



 **Supporting Fig. S5** Chemical structures of the compounds identified in the 78 species from the Lychnophorinae subtribe leaf extracts.

**

 Supporting Fig. S6** Chemical structures of the compounds identified in the *Lychnophora* species leaf extracts.

**

 Supporting Fig. S7** Chemical structures of the compounds identified in the *Eremanthus* species leaf extracts.
